# The RNA-Binding Protein ATXN2 is Expressed during Megakaryopoiesis and May Control Timing of Gene Expression

**DOI:** 10.3390/ijms21030967

**Published:** 2020-01-31

**Authors:** Marten Hansen, Sabrina Zeddies, Marjolein Meinders, Franca di Summa, Ewa Rollmann, Floris P.J. van Alphen, Arjan J. Hoogendijk, Kat S. Moore, Melanie Halbach, Laura Gutiérrez, Maartje van den Biggelaar, Daphne C. Thijssen-Timmer, Georg W.J. Auburger, Emile van den Akker, Marieke von Lindern

**Affiliations:** 1 Department Hematopoiesis, Sanquin Research, and Landsteiner Laboratory, Amsterdam University Medical Centre, 1066CX Amsterdam, The Netherlands; m.hansen@sanquin.nl (M.H.); sabrina.zeddies@gmx.de (S.Z.); f.disumma@sanquin.nl (F.d.S.); k.s.moore113@gmail.com (K.S.M.); d.thijssen@sanquin.nl (D.C.T.-T.); e.vandenakker@sanquin.nl (E.v.d.A.); 2 Department Blood Cell Research, Sanquin Research and Landsteiner Laboratory, Academic Medical Centre, University of Amsterdam,1066CX Amsterdam, The Netherlands; m.meinders@bristol.ac.uk (M.M.); l.gutierrez@me.com (L.G.); 3 Experimental Neurology, Goethe University Medical School, 60528 Frankfurt am Main, Germany; ewa.rollmann@gmx.de (E.R.); melanie.halbach@gmx.de (M.H.);; 4 Department of Molecular and Cellular Hemostasis, Sanquin Research, 1066CX Amsterdam, The Netherlandsa.hoogendijk@sanquin.nl (A.J.H.); m.vandenbiggelaar@sanquin.nl (M.v.d.B.)

**Keywords:** MEG-01, megakaryopoiesis, RBP, ATXN2, ATAXIN2, mRNA, translation, ITGB3, PECAM1, hemostasis

## Abstract

Megakaryopoiesis is the process during which megakaryoblasts differentiate to polyploid megakaryocytes that can subsequently shed thousands of platelets in the circulation. Megakaryocytes accumulate mRNA during their maturation, which is required for the correct spatio-temporal production of cytoskeletal proteins, membranes and platelet-specific granules, and for the subsequent shedding of thousands of platelets per cell. Gene expression profiling identified the RNA binding protein ATAXIN2 (ATXN2) as a putative novel regulator of megakaryopoiesis. *ATXN2* expression is high in CD34^+^/CD41^+^ megakaryoblasts and sharply decreases upon maturation to megakaryocytes. ATXN2 associates with DDX6 suggesting that it may mediate repression of mRNA translation during early megakaryopoiesis. Comparative transcriptome and proteome analysis on megakaryoid cells (MEG-01) with differential *ATXN2* expression identified ATXN2 dependent gene expression of mRNA and protein involved in processes linked to hemostasis. Mice deficient for Atxn2 did not display differences in bleeding times, but the expression of key surface receptors on platelets, such as ITGB3 (carries the CD61 antigen) and CD31 (PECAM1), was deregulated and platelet aggregation upon specific triggers was reduced.

## 1. Introduction

Megakaryopoiesis encompasses the commitment of hematopoietic stem cells (HSCs) towards megakaryoblasts (MKBLs) that proliferate and subsequently differentiate to large polyploid megakaryocytes (MKs), ultimately releasing platelets (PLTs) into the blood stream [1,2]. Megakaryopoiesis entails several unique features, including endomitosis [3]. These endomitotic cycles result in the serial duplication of the DNA content of the cell without subsequent cytokinesis, resulting in mature MKs containing a polylobulated nucleus that can reach up to 128N, and enlarged cytoplasm [3,4,5]. Although the expression of key transcription factors is not altered in MKs, expression of genes coding for platelet specific glycoproteins and granules increases [6]. The increase in nuclear material enhances mRNA synthesis capacity, and the increased cell size allows for the production of sufficient proteins and lipid membranes required to produce high quantities of PLTs [7]. Many of the proteins synthesized from this increased mRNA pool are packaged into the various granules released from PLTs upon activation [8]. When MKs reorganize their cytoskeleton during proplatelet formation, the granules are actively accumulated in the newly formed proplatelets. Ultimately, proplatelets are shed into the bloodstream where they circulate and mature into PLTs [9,10]. Upon vascular damage, PLTs interact with the exposed proteins of the subendothelial lining. This triggers signaling cascades, resulting in the activation and subsequent adhesion and spreading of PLTs at the site of damage, which promotes the local coagulation cascade in order to form a stable thrombus and fibrin plug. [11]. 

To understand the mechanism of MKs differentiation including protein production and granule synthesis, we performed gene expression profiling in human megakaryocytic cells (HaemAtlas) [12]. Among putative novel regulators of megakaryopoiesis we detected the RNA binding protein ATAXIN2 (ATXN2). 

ATXN2 [13,14] is a 140 kDa protein that has mainly been studied as a polyglutamine repeat protein in the context of spinocerebellar ataxia type 2 (SCA2) [15] and amyotrophic lateral sclerosis (ALS) [16,17,18,19]. It is part of the LSm family, and thereby linked to RNA processing and metabolism. Genome-wide association studies revealed single nucleotide polymorphisms in the *ATXN2* locus to be associated with an increased risk for thrombotic antiphospholipid syndrome or autoimmune disease [16,20,21].

Considerable progress has been made deciphering the mechanism involved in aggregate formation of polyQ-expanded ATXN2 protein in neurodegenerative disease, but the function of non-CAG repeat expanded ATXN2 remains elusive [22]. Several studies indicate that ATXN2 is involved in regulating mRNA stability and translation [23]. First, structural and functional analysis revealed domains involved in mRNA binding and translational regulation [24,25]. Next, ATXN2 has been described to associate with stress granules [26], the rough endoplasmic reticulum [27] and polyribosomes [28]. Lastly, ATXN2 was reported to promote microRNA-mediated mRNA breakdown [29,30]. In addition to a role in mRNA stability and translation, ATXN2 may control receptor endocytosis, actin filament formation and protein exocytosis [31,32,33,34].

Here, we show how ATXN2 affects the megakaryoid transcriptome and proteome. ATXN2 depletion resulted in deregulation of processes involved in platelet function and hemostasis. PLTs derived from Atxn2-deficient mice were characterized by increased expression of CD31 (Pecam1), more variable expression of other platelet surface markers and reduced aggregation upon specific triggers via the αIIβ3 (CD41(ITGA2B)/CD61(ITGB3)).

## 2. Results

### 2.1. ATXN2 Deficiency Does Not Alter Hematopoietic Lineage Commitment In Vitro

**Figure 1:**
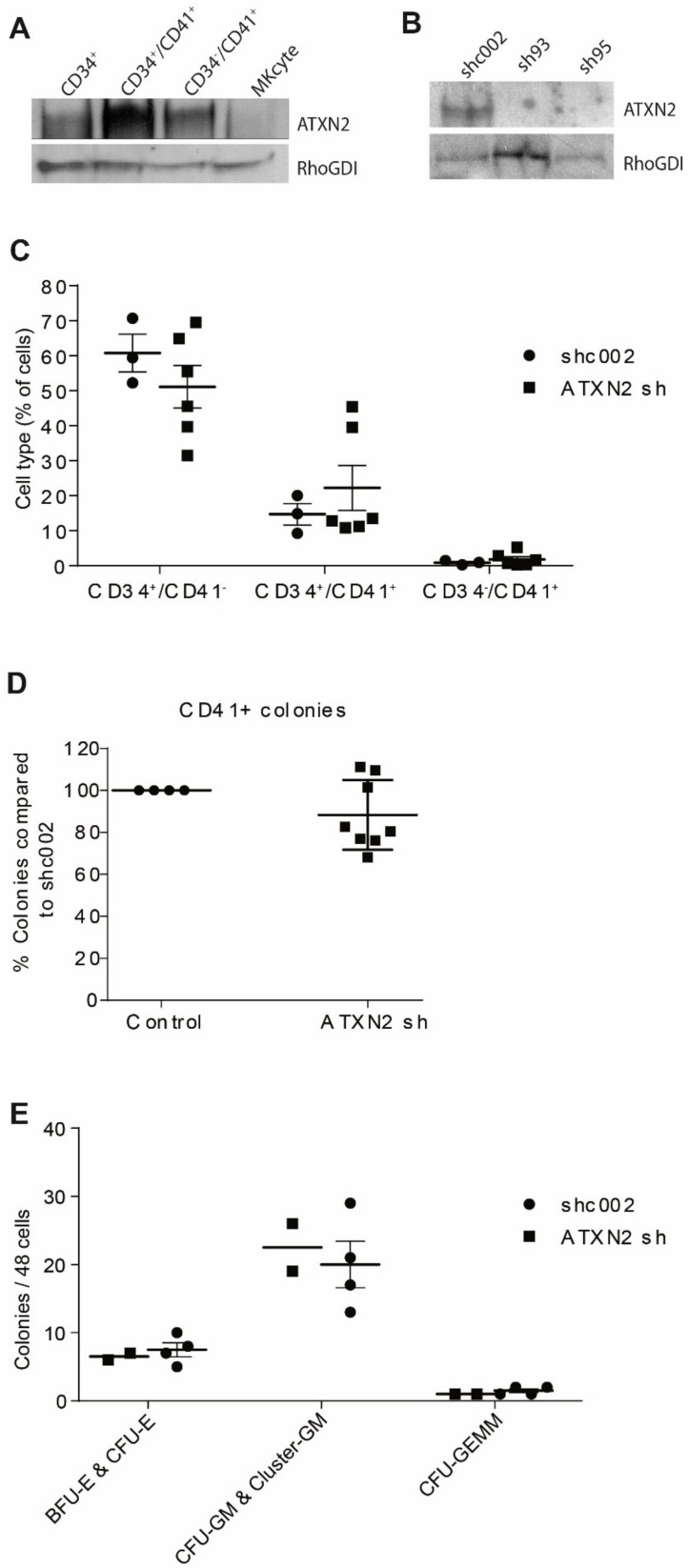
ATXN2 expression during megakaryopoiesis (**A**) Western Blots containing lysates of cells that represent different stages of megakaryopoiesis were stained for ATXN2 and RhoGDI (loading control). CD34^+^: uncultured cells, CD34^+^/CD41^-^ and CD34^+^/CD41^+^: sorted from day 7 MKs differentiation cultures, and CD34^-^/CD41^+^: harvested after an additional 7-day culture. (**B**) CD34^+^ cells were transduced with lentiviral vectors expressing green fluorescent protein (GFP) and shRNA directed against ATXN2 (sh93 or sh95) or control shRNA (sh002). GFP positive cells were sorted 48hours after transduction and cultured for three days. ATXN2 and RhoGDI expression was analysed in total cell lysates. (**C**) CD34^+^ cells, were transduced with shc002 (control shRNA), sh93, or sh95 (taken together as ATXN2 sh) and cultured for 5 days. Expression of CD34 and CD41 was assessed by flow cytometry, *n* = 3. (**D**) CD34^+^ cells were transduced with shc002, sh93 or sh95 and seeded into semisolid medium promoting megakaryocytic colony formation. After two weeks, CD41^+^ colonies were counted, shc002 set to 100%, *n* = 3. (**E**) Cells were transduced with shc002, sh93 or sh95 and single cell sorted into single well with semisolid medium. After two weeks, the amount of burst forming unit erythroid (BFU-E), colony forming unit erythroid (CFU-E), colony forming unit granulocyte macrophage (CFU-GM), and colony forming unit granulocyte, erythrocyte, macrophage (CFU-GEMM) were counted, *n* = 2 (ATXN2 sh) or *n* = 4 (control). Horizontal dash and error bars in **C**–**E** indicate mean and standard deviation.

To investigate the role of ATXN2 in megakaryopoiesis, we first determined the physiological expression of ATXN2 protein in distinct stages of human MKs development, from mobilized peripheral blood (MPB), that we defined as: CD34^+^/CD41a^−^ hematopoietic stem and progenitor cells (HSPC), CD34^+^/CD41a^+^ MKBLs, and committed maturing CD34^−^/CD41a^+^ MKs. ATXN2 expression increased from CD34^+^/CD41a^−^ to CD34^+^/CD41a^+^ MKBLs and sharply decreased again during differentiation to CD34^−^/CD41a^+^ MKs (Figure 1A). Next, we used shRNA to deplete *ATXN2* in CD34^+^ HSPC that were subsequently cultured for 5 days towards the megakaryocytic lineage. Two shRNA directed against *ATXN2* (sh93 and sh95) greatly reduced ATXN2 protein expression in CD34^+^ HSPC compared to control shRNA (shc002) (Figure 1B). Knockdown of *ATXN2* did not affect the distribution of CD34^+^ HSPC, CD34^+^/CD41a^+^ MKBLs, and CD34^−^/CD41a^+^ mature MKs compared to shc002 (Figure 1C). CD34^+^ cells transduced with *ATXN2* shRNA or control shRNA gave rise to similar numbers of CD41a^+^ megakaryocytic colonies when seeded in semisolid medium (MegaCult) (Figure 1D). In addition, we observed no difference in the distribution of burst-forming unit erythroid (BFU-E), colony-forming unit erythroid (CFU-E), colony forming unit granulocyte macrophage (CFU-GM) and colony forming unit granulocyte, erythrocyte, monocyte, macrophage (CFU-GEMM) between cells transduced with shc002 or *ATXN2* shRNA (Figure 1E). Taken together, loss of ATXN2 did not influence in vitro hematopoietic lineage fate or early megakaryocytic differentiation. 

### 2.2. ATXN2 Associates with DDX6 and PABP in MKs

**Figure 2:**
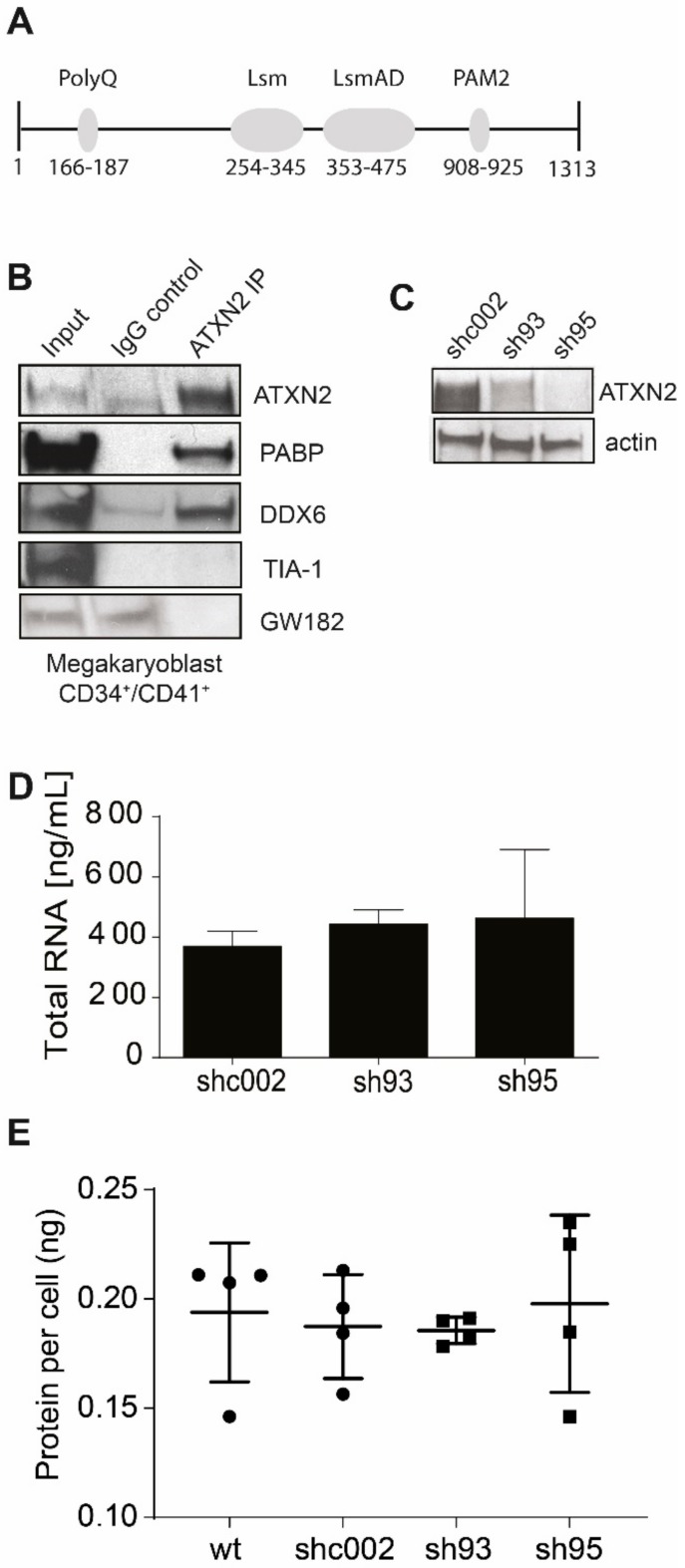
ATXN2 associates with proteins implicated in mRNA storage and decay. (**A**) Schematic representation of functional domains within ATXN2, positions indicate amino acids. PolyQ (polyglutamine tract), Lsm (Like Sm), LsmAD (Like Sm associated domain), PAM2 (poly(A)-binding protein interacting motif). (**B**) Immunoprecipitation (IP) using ATXN2 antibody (ATXN2 IP) or control antibodies (IgG control) in CD34^+^/CD41^+^ MKBLs. Input is cell lysate before IP. Membranes were probed for PABP, DDX6 and TIA-1 and GW182. (**C**) Western blot of total cell lysate, harvested 3 days after transduction of MEG-01 cells with lentiviral vector expressing shRNA (shc002 control or ATXN2 sh93 or sh95), stained for ATXN2 and actin (loading control), representative image, *n* = 5 (**D**) RNA content measured in MEG-01 cells after *ATXN2* depletion (day 7) compared to control cells (shc002), *n* = 5. (**E**) Protein content in 10^6^ MEG-01 cells in absence and presence of *ATXN2* (day 16; control indicates non-transduced MEG-01), *n* = 4. Horizontal dash and error bars in C-E indicate mean and standard deviation.

During MKs polyploidization the cells expand in volume as they produce granules and the extensive demarcation membrane system (DMS), a prerequisite to produce thousands of PLTs per MK. This process is associated with marked changes in protein expression. ATXN2 contains several domains facilitating binding to both mRNA and proteins to function in mRNA translation depicted in Figure 2A [24,26,28,35]. We first investigated if interactions with known binding partners occurs in megakaryocytic cells. Immunoprecipitations demonstrated association of ATXN2 with poly(A) binding protein (PABP) and the RNA helicase DDX6 in primary human MKBLs (Figure 2B). The DDX6-PABP complex is involved in miRNA-mediated mRNA decay [36]. ATXN2-associated DDX6/PABP-bound mRNA, however, is silenced and not degraded in P-bodies [37]. Accordingly, also in megakaryocytic cells, ATXN2 did not associate with the P-body specific proteins GW182 (an RNAse and marker for P-bodies) and TIA1 (an RNA-binding protein and marker for stress granules) (Figure 2B). Because the association of ATXN2 with DDX6 predicts repression of mRNA translation, we next investigated whether loss of ATXN2 impacts on general protein synthesis. MEG-01 cells were transduced with the same shRNA constructs used for CD34^+^ HSPC (sh93 and sh95 against *ATXN2,* shc002 control) (Figure 2C). Seven days following transduction, there was no change in RNA content upon *ATXN2* loss (Figure 2D), and we also did not observe an effect on total protein content (Figure 2E). MEG-01 cells with or without ATXN2 expression displayed similar proliferation dynamics.

### 2.3. Megakaryocytic mRNA and Protein Homeostasis Regulated by ATXN2

**Figure 3:**
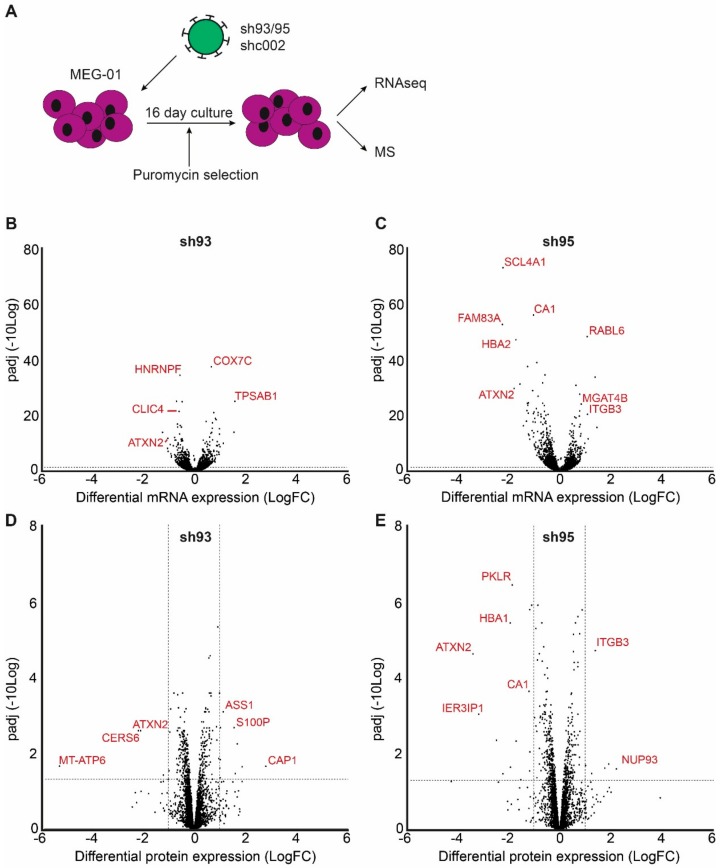
Deregulation of RNA and protein content in MEG-01 cells after ATXN2 depletion (**A**) Schematic representation of MEG-01 transduction with lentiviral vectors expressing shc002 control, or *ATXN2*-directed sh93 or sh95 shRNA. Transduced cells were selected for puromycin resistance throughout the culture period. Cells were harvested 16 days post-transduction for parallel RNA sequencing and mass spectrometry analysis. (**B–E**) Differentially expressed genes (FDR < 0,05, *n* = 3) between shc002 and sh93 (**B**,**D**) or sh95 (**C**,**E**) transduced cells were identified on mRNA (B, C) and protein (**D**,**E**) level. A Differential expression (log2 fold change) is plotted against the adjusted p value (padj transformed as –log10 values). A selection of genes is marked.

We next investigated whether ATXN2 controls stability or translation of specific mRNAs during megakaryopoiesis. ATXN2 expression was knocked down by shRNA in MEG-01 and samples were harvested 16 days following shRNA transduction for parallel mass spectrometry and RNA-sequencing (Figure 3A). 

Parallel samples were processed to generate expression profiles of poly-adenylated mRNA (*n* = 3 per condition). Following normalization, relative mRNA levels were calculated as Transcripts Per Kilobase Million (TPM). Desq2 analysis was performed to identify genes with differential mRNA expression between samples transduced with sh93 or sh95, and samples transduced with sh002 (FDR < 0.05) (Figure 3B,C) [38]. In total, 1255 and 1842 genes were differentially expressed upon *ATXN2* depletion, for sh93 and sh95 respectively, with an overlap of 454 genes (Supplemental Tables S1 and S2). Note that ATXN2 is among the significantly downregulated RNA’s confirming the knockdown using shRNAs. 

**Figure 4:**
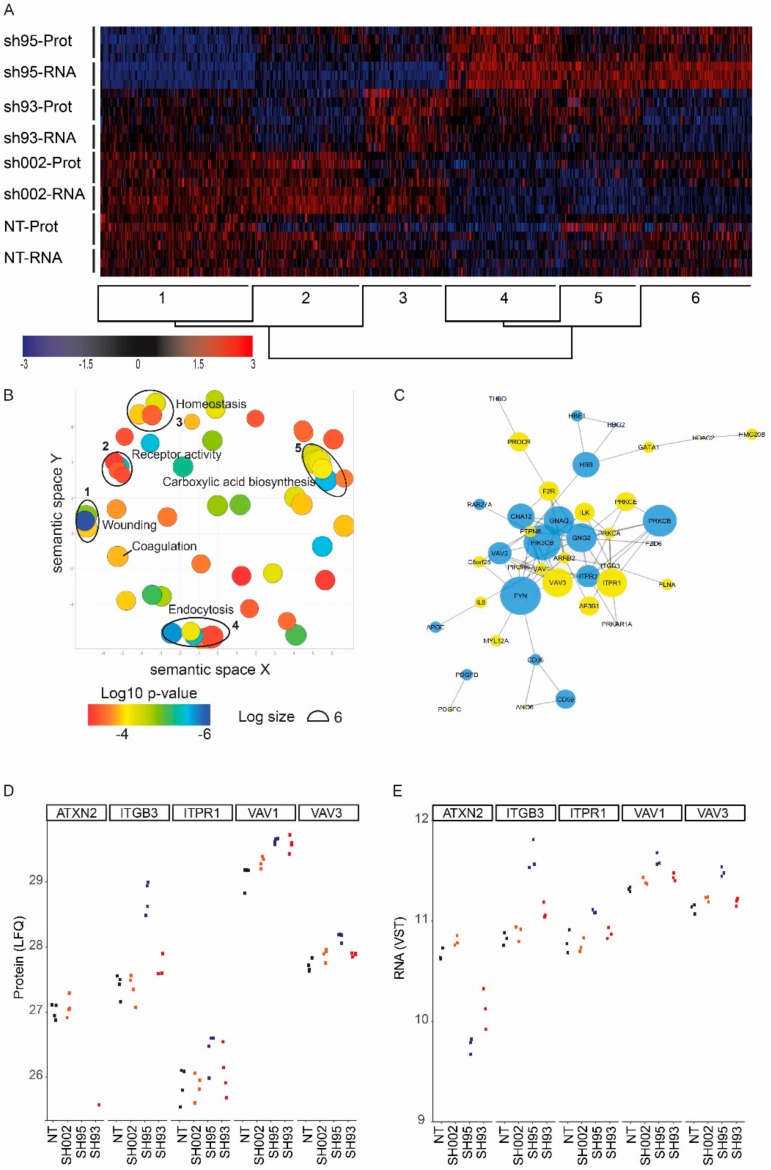
Gene ontology analysis links ATXN2 to platelet-related or haemostasis pathways. (**A**) Hierarchical clustering of genes that are differentially expressed at the RNA level between sh002 (control) and sh95 transduced MEG-01 cells (FDR < 0,05) and significantly detected by mass spectroscopy. (row clusters, k = 6, blue = downregulated, red = upregulated). Genes are listed in Supplemental Table S2. These genes were subjected to GO Term enrichment analysis. (**B**) Deregulated processes upregulated upon ATXN2 depletion were visualized with REVIGO, clustering similar GO terms together in a semantic space (full list of GO terms in Supplemental Table S5). Closely related GO terms were encircled, numbers correspond to column “RIVIGO” in Supplemental Table S2. (**C**) STRING analysis of deregulated genes associated to the GO term coagulation, blue downregulated and yellow upregulated genes. Node size corresponds with known interactions (more = larger) with other proteins within the cluster. (**D,E**) Expression level of ATXN2 and a selection of genes that are associated with platelet function in non-transduced cells (NT), sh002 control, sh95 or sh93 transduced MEG-01 cells measured by (**D**) label-free quantification (LFQ) of protein and (**E**) variance-stabilizing transformed (VST) mRNA expression.

Cell lysates that were harvested in parallel with the RNA samples were subjected to mass spectrometry. Differential protein abundancies were determined with moderated t-test using LIMMA. (Figure 3D,E). In accordance with the efficient knock down in CD34^+^ HSPC (Figure 2C), we did not find ATXN2 peptides in cells transduced with sh95, while in sh93 ATXN2 peptides (1/4) were observed but to a significantly lower amount compared to control (Figure 4A,D). Because sh95 gave a stronger reduction in ATXN2 protein and mRNA expression we focused on differential protein expression in sh95 transduced MEG-01 cells. Differential expression was calculated with an FDR of <0.05 and an absolute log fold change >1 (Figure 3C, Supplemental Table S3). In contrast to large numbers of differentially expressed transcripts, only 20 proteins were differentially expressed at this statistical threshold. Although we detected >1.000 genes on mRNA level that are differentially expressed, this is often buffered at the protein level, on which we detected 631 of these differentially expressed RNA genes [39,40,41,42,43]. Comparison of differential mRNA expression with the detected proteins indicated that the protein of many differentially expressed transcripts are not significant and/or detected (data not shown). To compare mRNA and protein expression and their regulation between the different samples, we combined the Z-transformed expression data of differentially expressed transcripts (sh95 versus sh002) with Z-transformed expressed proteins in a heatmap with hierarchical ordering of RNA and protein samples in columns, and rows cluster in 6 groups (k = 6, Figure 4A). Only cluster 4 and 6 show upregulation of transcripts with similar protein expression comparing sh002 control and ATXN2 suppression by sh95, suggesting ATXN2 promotes protein expression of genes represented in this cluster (Supplemental Table S4). The comparison also showed that the two short hairpins used to reduce ATXN2 induced similar up- or downregulation with shRNA95 showing increased deregulated expression compared to shRNA93 which is in accordance with the knockdown levels. 

To identify cellular processes regulated by ATXN2, we analyzed which GO terms were enriched among differentially expressed transcripts (sh002 versus sh95-treated MEG01 cells). Enriched GO terms were visualized with REVIGO [44]. Amongst the identified affected processes an ordering on semantic space reveals clustering of multiple enriched and related GO terms, e.g coagulation and wounding (Figure 4B, Supplemental Table S5). GO terms are very useful indicators of affected processes, but not always refer to specific processes. “Coagulation and wounding englobe a number of proteins/genes which are either related to the coagulation cascade itself, but also all proteins/genes that have been described as platelet- or megakaryocytic-specific, given the role of platelets in general hemostasis. To get more insight, we further performed string analysis on the GO term “coagulation” enriched proteins/genes to uncover implicated regulators and visualize differential expression (Figure 4C). This uncovered a network of interactions of terms directly related to platelet physiology, including calcium sensitive genes involved in platelet activation. Furthermore, also regulation of receptor activity was also amongst deregulated terms, as we found multiple cell surface receptors to be deregulated including CD36, CD56, ITGB3, F2R, C6orf25 (MPIG6B). From these, especially ITGB3, F2R(PAR-1) and C6orf25 are of particular interests because these are directly implicated in platelet aggregation [45,46,47]. Most of these receptors were, however, not detected by mass spectroscopy and are not represented in the heatmap except for ITGB3 (cluster 4; Figure 4A). In addition to the receptors, multiple downstream signaling intermediates involved in platelet function were differentially expressed. Among those were ILK (Integrin Linked Kinase), VAV1 and VAV3 (cluster 4; Figure 4A), AP3B1 (Adaptor Related Protein Complex 3 Subunit Beta 1), and PTPN6 (Protein Tyrosine Phosphatase Non-Receptor Type 6, also known as SHP1) (cluster 5, Figure 4A; Supplemental Table S4). 

RNA and protein expression of a small selection of these genes was compared between two levels of ATXN2 knock down (by sh95 and sh93). The level of knock down of ATXN2 corresponded to the level of deregulated gene expression (Figure 4D,E).

### 2.4. Atxn2 Affects Megakaryopoiesis on the MKBLs Stage In Vivo

**Figure 5:**
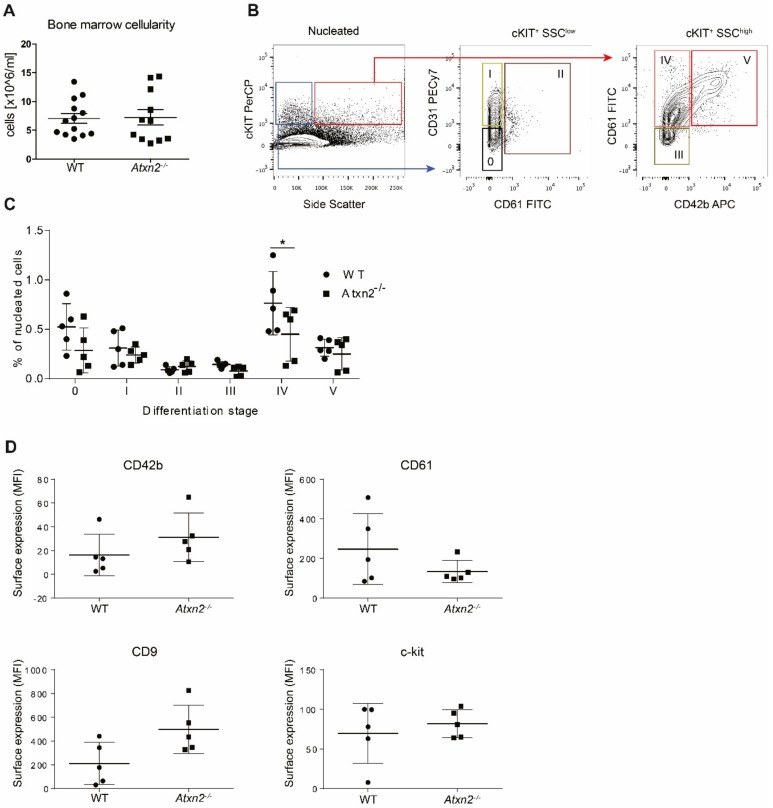
ATXN2 deficiency alters early megakaryocytic differentiation. (**A**) Femurs of *WT* or *Atxn2^−/−^* mice were crushed, and bone marrow cellularity (recovered nucleated cells) per femur was determined *n* = 8 (**B**) Stages 0, I and II of megakaryopoiesis were characterised based on cKit expression and low side scatter (blue box, first panel) and subsequently divided in the 3 stages based on CD31 and CD61 expression (second panel). Stages III, IV and V were characterised based on cKIT expression together with high side scatter (SSC^high^)(red box first panel) and subsequently on CD61 and CD42b expression (third panel). The percentage of cells in each stage was calculated as a percentage of all nucleated cells measured in the first panel. (**C**) Percentage of different MKs differentiation stadia in *WT* or *Atxn2^−/−^* mice femurs, * *p* < 0.05. (**D**) Expression of MKs surface proteins CD42b, CD61, CD9 and c-Kit measured by flow cytometry on cells isolated from femurs of *WT* and *Atxn2^−/−^* mice. Expression is given as mean fluorescent intensity (MFI) in arbitrary units. *n* = 5.

To further examine the function of ATXN2 in megakaryopoiesis and thrombopoiesis, we investigated aspects of megakaryopoiesis and platelet function in *Atxn2* deficient mice (*Atxn2^−/−^*). These mice present with smaller litters, a segregation distortion with more male than female offspring and increased body weight [48,49]. *WT* and *Atxn2^−/−^* mice yielded similar numbers of total BM cells (Figure 5A). Megakaryopoiesis was assessed by side scatter distribution and surface marker expression, which allows to discern 5 stages (Figure 5B) [50]. This showed a decrease of stage IV MKs within the nucleated cell fraction in *Atxn2^−/−^* mice (Figure 5C). Stage IV corresponds with human MKBLs, in which we found high ATXN2 expression (Figure 1A). No significant differences in the cell surface expression of the megakaryocytic/hematopoietic markers CD42b, CD61, CD9 and c-kit was detected on BM MKs, although an upward trend was visible for CD42b, CD9 and a downward trend for CD61 (Figure 5D). 

### 2.5. Atxn2 Deficiency Causes a Platelet Aggregation Defect In Vitro

**Figure 6:**
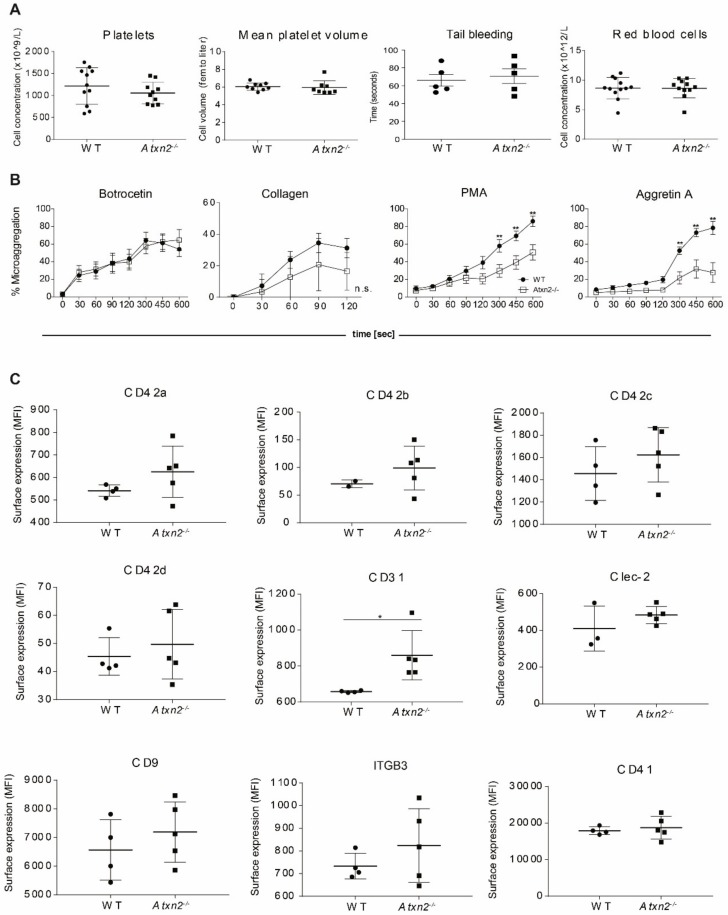
ATXN2 deficiency causes a platelet aggregation defect. (**A**) Concentration of platelets and platelet size, tail bleeding time and concentration of red blood cells in blood obtained by retroorbital sampling from *WT* and *Atxn2^−/−^* mice, *n* = 8. (**B**) To measure aggregation of PLTs from *WT* or *Atxn2^−/−^* mice, PLTs were isolated and split into two separate tubes, labelled with different fluorochromes, and mixed. PLTs were activated with PMA (100 ng/ml), Aggretin (300 µM), botrocetin (10 µg/ml) or collagen (10 µg/ml) and the increase in double labelled aggregates (percentage) over time was measured by flow cytometry, *n* = 5, ** *p* < 0.01. (**C**) Platelet receptor surface expression measured with flow cytometry on *WT* or *Atxn2^−/−^* PLTs in resting state, expression level indicated as mean fluorescent intensity (MFI), *n* = 5, * *p* < 0.05.

Because the RNA-seq and proteomics data suggest an unbalanced expression of components of the ITGB3 signaling pathway in megakaryoid cells, we evaluated platelet function in *Atxn2^−/−^* and *WT* mice. Bleeding times were similar. Platelet count, mean platelet volume as well as red and white blood cell counts were not significantly changed between *Atxn2^−/−^* and *WT* animals (Figure 6A). To measure the efficiency of platelet aggregation, PLTs from *Atxn2^−/−^* and *WT* mice were activated with four specific agonists and microaggregation was measured by flow cytometry following a time course as described [51]. Aggregation of Atxn2^−/−^ and WT PLTs was comparable upon addition of botrocetin, which induces signaling via the von Willebrand Factor (vWF) receptor complex containing the glycoproteins (GP) GPIBA and GPIBB (CD42b and CD42c), GPV (CD42d), and GPIX (CD42a), or collagen, which binds GPVI and integrin αIIβI. In contrast, aggregation of *Atxn2^−/−^* PLTs was reduced 2-fold compared to *WT* PLTs upon addition of the αIIbβ3 integrin (ITGA2B or CD41 and ITGB3 or CD61) agonist PMA and almost 4-fold in response to the Clec-2 agonist Aggretin A, which induces platelet aggregation via intracellular activation of the αIIbβ3 integrin. This suggests that both αIIbβ3 integrin ouside-in and inside-out signaling is compromised in Atxn-/- platelets, albeit αIIbβ3-dependent aggregation was not completely ablated (Figure 6B). *Atxn2^−/−^* PLTs presented still fully functional responses via the vWF (agglutination) and collagen (aggregation) receptors, compared to WT. To investigate whether the unbalanced aggregation profile of Atxn2-/- platelets could be due to dysregulated receptor surface expression, we analyzed resting PLTs from *Atxn2^−/−^* and *WT* mice by flow cytometry for expression of the most abundant platelet receptors. Expression of all tested antigens was mostly similar in *Atxn2^−/−^* PLTs compared to *WT*, although subject to variation (CD42 and ITGB3). Only CD31 expression was 30% increased (Figure 6C). Increased CD31 expression has been implicated in aberrant platelet aggregation, as it affects αIIbβ3 integrin stimulation [52]. 

## 3. Discussion

The burst of platelet production by MKs requires that the production of platelet and thereby platelet proteins, is well controlled in space and time. Previous transcriptome analysis revealed a putative novel regulator of megakaryopoiesis: the RNA binding protein ATXN2. Here we showed that ATXN2 is expressed in MKBLs and downregulated upon terminal MKs differentiation. ATXN2 is associated with DDX6 and PABP, but not with the P-body specific proteins TIA-1 and GW182 that direct mRNA degradation, which suggests that ATXN2 may repress mRNA translation as described in other cell types. Depletion of *ATXN2* in the MKBLs cell line MEG-01 deregulated mRNA expression of genes important for platelet physiology, without major effect on protein composition. Mice lacking *Atxn2* exhibit a decrease in type IV megakaryoid cells, which corresponds to the cell type with the highest *ATXN2* expression in vitro. Despite this decrease in type IV megakaryoid cells, the next stage of maturation seems unaffected by the level of Atxn2, at least in cell abundance. This could be the effect of feedback control at the other stages of megakaryopoiesis. It could also be that Atxn2 deficiency causes rapid transition through stage IV and subsequent increased proliferation in stage V. Better insight into stage specific effects of Atxn2 is required to explain the observed change in cell frequencies. Premature expression of late megakaryoid proteins may enhance maturation of stage IV cells and thereby reduce the relative number of stage IV cells. The reduced αIIbβ3-mediated platelet aggregation observed in ATXN2 deficient mice may be caused by an unbalanced expression of functional proteins among which key signaling molecules, as we were able to show that αIIbβ3 integrin mediated platelet aggregation is impaired upon stimulation with PMA (outside-in signaling) or with Aggretin A (inside-out signaling, via CLEC2 receptor activation). 

ATXN2 binds AU-rich elements in the 3’ UTR of mRNA, and interacts with the RNA-binding proteins PABP and DDX6 [22,36,37]. This prompted us to investigate the effect of ATXN2 on mRNA and protein expression. We used the MEG-01 megakaryoblast cell line in which after 16 days analyzed for transcriptional and protein alternation. The advantage of this model is the abundant availability of cells that are a relative homogenous population compared to primary cells and the possibility to select for cells expressing the knock down vectors. We realize our approach does not cover the effects on megakaryocytes and the aspects of thrombopoiesis in this setting. In addition, the selection process and the analysis at day 16 post transduction imply that we examine cells in which gene expression is affected as a response to changes in ATXN2 target genes. Many targets may be indirect targets, regulated at transcriptional level and not at mRNA stability or translation. Thus, it is not surprising that mRNA and protein expression do not mirror each other.

The function of many differentially expressed transcripts is associated with wound healing, platelet function, and coagulation (as referred to in GO Term databases). Moreover, many of these genes are upregulated following ATXN2 knock down. This supports the hypothesis that ATXN2 suppresses the expression of proteins present in PLTs. Given the observation that ATXN2 is abundantly expressed in megakaryoblasts, the MEG-01 may be the correct cell stage to probe ATXN2 function. Identification of direct targets of ATXN2 in megakaryoblasts would require precipitation of ATXN2-RNA complexes, which was beyond our possibilities. 

Bleeding time and platelet numbers were unaffected in Atxn2-deficient mice. Firstly, bleeding times may be more dependent on fibrin clot formation, and platelet numbers are restricted by the availability of TPO (thrombopoietin). Feedback control between the number of PLT and TPO production will retain a proper balance when PLT formation is disturbed. The function of PLT was altered in Atxn2-deficient mice. Platelet aggregation in response to specific agonists was reduced, mainly upon Aggretin A, which induces aggregation via CLEC2 and subsequent αIIbβ3 activation. The αIIbβ3 integrin receptor is a heterodimer between ITGA2B and ITGB3, the latter being upregulated at protein and mRNA level when *ATXN2* is depleted. However the surface expression of CD41 and ITGB3 measured by flow cytometry did not differ between *WT* and *Atxn2*^−/−^ PLTs. Controlled receptor transport to the cell surface may maintain ITGB3 cell surface expression even at higher intracellular expression levels. In contrast, CD31 surface receptor expression increased significantly. It has been previously shown that clustering of CD31 negatively influences platelet aggregation upon activation by ADP, cross-linked collagen and thrombin [52,53]. However, we did not detect altered collagen responses. Together, this may indicate that deregulation of intracellular signaling rather than receptor levels may underly the aggregation properties of platelets in Atxn2-deficient mice. Among the molecules implemented in the GO term “coagulation” that were up (32) down (22) regulated were F2R, VAV1,C6orf25 and ITGB3, which are relevant for platelet physiology [45]. Thus, aberrant surface receptor expression of CD31 and deregulated total expression of ITGB3 could lead to a decreased aggregation in response to PMA and Aggretin A, in conjunction with C6orf25, F2R and VAV1 dysregulation, that are needed for the response to these stimuli. 

## 4. Conclusions

Loss of Atxn2 affects the platelet aggregation capacity profile, however, general hemostasis is unaffected. ATXN2 contributes to megakaryopoiesis during the MKBLs stage when mRNA translation needs to be repressed until further maturation towards MKs is initiated. Depletion of ATXN2 leads to minor deregulations in protein expression, which affects signaling proteins in PLTs, and thereby their aggregation potential The role of ATXN2 in the regulation of homeostasis is subtle, but alterations in ATXN2 expression may be directly involved in hemostasis.

## 5. Methods

### 5.1. Analysis of Mouse Blood and Bone Marrow

Blood was drawn from Atxn2 knockout (*Atxn2^−/−^)* ^33^. and wildtype (*WT*) animals by retroorbital puncture using heparin-coated glass capillaries (Hirschmann, Eberstadt, Germany), and collected in heparin-coated vials. Blood parameters were determined on a scil Vet abc Plus+ instrument (scil animal care, Oostelbeers, the Netherlands). Platelet rich plasma (PRP) was separated by centrifuging the blood 15 min at 50 g. To analyze platelet aggregation, PLTs were split into two separate tubes, labelled with different fluorochromes, mixed. Samples were analyzed by flow cytometry for association of fluorochromes as described [51]. 

BM was harvested from mice anesthetized using Isofluran (Baxter, Unterschleissheim, Germany) and sacrificed by perfusion with 4% paraformaldehyde. This procedure was followed because other tissues were analyzed for independent studies. Following perfusion, femurs were isolated, crushed and bone marrow (BM) cells were resuspended in PBS + 0.1% HSA. BM suspensions were passed through a cell strainer (VWR, Amsterdam, the Netherlands) and counted on a Casy Cell Counter (Roche, Woerden, the Netherlands). Cells from blood or BM were stained with cMpl (Santa Cruz, Heidelberg, Germany), Clec-2 (AbD Serotec, Pucheim, Germany), CD9-PE (Abcam, Cambridge, GB), CD61-FITC, CD41-PE, KIT PerCPCy5.5, (BD Biosciences, Allschwil, Switzerland); CD42a-FITC, CD42b-DL649, CD42c-FITC, CD42d-FITC (Emfret, Eibelstadt, Germany); CD31 PE-Cy7 (Abcam, Cambridge, UK) using a 1:1000 dilution for all antibodies. samples were measured using a flow cytometer (LSRII + HTS, BD Biosciences, Allschwil, Switzerland) and data were analyzed with FlowJo software version 9.2 (Tree Star Inc., Ashland, OR, USA). 

### 5.2. Human CD34^+^ Cultures and Cell Lines

MEG-01 were cultured in RPMI 1640 (Gibco) supplemented with 20% FSC, 50 U/mL penicillin-streptomycin, L-glutamin 2 mM (ThermoFisher Scientific, USA). Mobilized peripheral blood was provided by the Sanquin Laboratory for Cell Therapy and obtained from leukopheresis material of healthy donors treated with G-CSF (2 × 5 µg/kg/day subcutaneously, Filgastrim, Amgen, Breda, The Netherlands) as described [54]. Informed consent was given in accordance with the Declaration of Helsinki and the Dutch national and Sanquin internal ethical review boards. For MKs cultures, CD34^+^ cells were cultured in CellGro medium (CellGenix, Frankfurt, Germany) supplemented with TPO (N-plate, Amgen, Breda, the Netherlands) and IL-1β (PeproTech, Heerhugowaard, the Netherlands). At designated time-points, cultures were sorted for CD34 and CD41 expression, using the antibodies specified above, on an Aria II cell sorter (BD Biosciences, Allschwil, Switzerland). For phenotypic analysis, cells were fixed with 1% PFA, washed with PBS + 0.05% BSA + 0.05M EDTA and incubated with CD34 Pe-Cy7, CD41 APC or CD42b APC (all BD Biosciences, Allschwil, Switzerland). Samples were measured with flow cytometry (LSRII + HTS, BD Biosciences, Allschwil, Switzerland). For ATXN2 expression level during megakaryopoiesis, cell were cultured in the presence of TPO and IL-1β and cell sorting was performed after 7 days of culture to obtain CD34^+^/CD41^-^ and CD34^+^/CD41^+^ fractions. Subsequent culture of the CD34^+^/CD41^+^ fraction resulted in a sample of terminally differentiated CD41^+^ cells.

### 5.3. Lentivirus Production and Lentiviral Transduction

The lentiviral knockdown vector SIN.PPT.CMV.GFP.U3*Nhe*1 and puro pLKO.1 was a kind gift of N.A Kootstra (Academic Medical Center, Amsterdam, The Netherlands) [55]. We cloned a cassette containing the short hairpin RNA (shRNA) sequence under the control of a U6 promoter. The shRNA sequences used against *ATXN2* were sh93: GCCAAGACATATAGAGCAGTA, sh95: CCGAAGTGTGATTTGGTACTT and as a non-targeting shRNA control (shc002) CAACAAGATGAAGAGCACCAA was used. All cloned short hairpins were verified by sequencing.

Lentiviral particles were produced in 293T cells using the third generation system and subsequent transduction of CD34^+^ PBMC or MEG-01 cells was carried out as described before [54]. CD34^+^ were then seeded into megakaryocytic liquid cultures or used for colony formation assays. Megacult colony formation assay (Stem Cell Technologies, Grenoble, France) and Colony Gel colony formation assay (Cell Systems, Frankfurt, Germany) were carried out according to the manufacturer’s specifications. MEG-01 for mass spectrometry and RNA sequencing were sorted 16 days after transduction, without prior fixation. 

### 5.4. Immunoprecipitation and Immunodetection

Immunoprecipitation was carried out on protein extracts from CD34^+^/CD41^+^ MKBLs. Cells were lysed in NP-40 buffer (20 mM Tris pH 8.0, 137 mM NaCl, 2 mM EDTA, 10% Glycerol, 1% NP-40), supplemented with protease inhibitor cocktail (Roche, Woerden, The Netherlands) and 25 U/ml benzonase (Merck, Darmstadt, Germany). 5 μl of ATXN2 antibody (BD Biosciences, Allschwil, Switzerland) or IgG control antibody (Sanquin, Amsterdam, The Netherlands) was added to the lysate and incubated overnight at 4 °C. The next day, sepharose beads (Pierce, Rockford, IL, USA) were added and incubated for at least 6 hours. Beads were then washed three times with NP-40 lysis buffer and eluted using SDS sample buffer. Samples were loaded on a 10% precast gel (Thermo Scientific, Waltham, MA, USA) and transferred onto nitrocellulose membranes using iBlot (Invitrogen, Bleiswijk, The Netherlands). Membranes were probed with ATXN2 (BD Biosciences, Allschwil, Switzerland), DDX6 (Novus Biologicals, Littleton, CO), PABP (Abcam, Cambridge, GB) and TIA-1 (Santa Cruz, Heidelberg, Germany). Secondary anti mouse-HRP antibody or anti rabbit-HRP antibody (both Dako, Glostrup, Denmark) was applied and membranes were developed by Enhanced Chemiluminescence (Pierce, Rockford, IL, USA).

### 5.5. Mass Spectrometry

Non transduced, sh002, sh93 and sh95 transduced MEG-01 cells cultured for 16 days after transduction were subjected to mass spectrometry (samples for RNA collected parallel). Eluted peptides were processed as described [56]. Samples were subjected to mass spectrometry using label-free quantification. All data was processed with MaxQuant for peptide identification and quantification [57]. Downstream statistical analysis was performed with R 3.5.3. All proteins matching the reverse database, potential contaminants, and those only identified by site were filtered out. To be considered for analysis, a protein had to be detectable within all replicates of at least one sample group (non-transduced and sh002 or sh93 and sh95). Prior to statistical analysis, a log2 transformation was performed. Missing values were imputed from the normal distribution with a width of 0.3 and a downshift of 1.8 [58]. Differential protein expression was determined using LIMMA for sh002 versus sh93 and sh95 with an adjusted *p* value of >0.05 and an absolute log fold change >1. 

### 5.6. RNA-Sequencing

Non-transduced, sh002, sh93 and sh95 transduced MEG-01 cells cultured for 16 days after transduction were subjected to RNA-sequencing (samples for mass spectrometry collected parallel). RNA expression by total mRNA sequencing was performed by Novogene Co., LTD (Cambridge, UK). Briefly, library preparation was performed using the NEB Next® Ultra™ RNA Library Prep Kit (New England Biolabs, Ipswich, MA, USA) and enriched using oligo(dT) beads. Isolated mRNA was fragmented randomly in fragmentation buffer, followed by cDNA synthesis using random hexamers and reverse transcriptase. After first-strand synthesis, a custom second-strand synthesis buffer (Illumina, San Diego, CA, USA) was added with dNTPs, RNase H and *Escherichia coli* polymerase I to generate the second strand by nick-translation. The final cDNA library is ready after a round of purification, terminal repair, A-tailing, ligation of sequencing adapters, size selection and PCR enrichment. The complete library was sequenced using Illumina HiSeq 2500 (2 × 150bp, paired end). Sequence quality was confirmed using Fastqc (Babraham Bioinformatics, Cambridge, UK). Spliced Transcripts Alignment to a Reference (Salmon [59]) was used to align the sequences to the human hg38 genomic reference sequence, using the following parameters: --outFilterMultimapNmax 20, --outFilterMismatchNmax 1, --outSAMmultNmax 1, -outSAMtype BAM SortedByCoordinates, quantMode GeneCounts, -outWigType wiggle, -outWigStrand Stranded, --outWigNorm RPM. A .gtf file accessed from the UCSC genome browser on August-2018 was passed to Salmon [59]. RNA expression levels were normalized using size factors calculated by DESeq2 [38].

### 5.7. Gene Ontology Term Enrichment Analysis

Enrichment of gene ontology (GO) terms based on biological processes, molecular functions and cellular components was performed with R package bioMaRt [60]. Visualization of significant GO Terms was done with the REVIGO web tool [44]. This summarizes GO terms by finding a representative subset of terms that relies on semantic similarity measures. Significant GO terms were plotted with their respected *p* values and an allowed similarity of 0.7.The full lists of GO Terms is provided in Supplementary Table S5. STRING analysis of significantly found genes within a GO Terms were with a confidence of 0.7 extracted from (string.org, version 11.0 [61]). Cytoscape 3.5.1 was used to visualize the interaction networks [62]. 

### 5.8. Data Accessibility

RNAseq raw data is accessible through https://www.ncbi.nlm.nih.gov/geo/ under GSE131308. Raw mass spectrometry data is accessible through ProteomeXchange with identifier PXD015823.

## Supplementary Materials

Supplementary materials can be found at https://www.mdpi.com/1422-0067/21/3/967/s1. Table S1: Normalized read counts RNAseq after 16 days of ATXN2 depletion in MEG-01. Table S2: related to Figure 3B,C. Fold change and *p*-value of sh93 and sh95 after ATXN2 depletion based on RNAseq. Table S3, related to Figure 3D,E, Figure 4D. Log2 transformed protein expression for detected genes by mass spectrometry. Table S4, related to Figure 4A. Z-scored protein and RNAseq expression, of significant genes of sh95 that were also detected on protein level. Table S5, related to Figure 4B. Full list of Go Terms that were differentially up and down regulated based on mRNA expression upon ATXN2 depletion with sh95 in MEG-01.
